# Prevalence and socio-economic factors affecting the use of traditional medicine among adults of Katikekile Subcounty, Moroto District, Uganda

**DOI:** 10.4314/ahs.v21i3.52

**Published:** 2021-09

**Authors:** Annie Logiel, Erik Jørs, Pardon Akugizibwe, Peder Ahnfeldt-Mollerup

**Affiliations:** 1 Clarke International University, Uganda; 2 Odense University Hospital, Department of Occupational and Environmental Medicine; 3 Odense University Hospital, The International Centre for Occupational, Environmental and Public Health (ICOEPH); 4 University of Southern Denmark Faculty of Health Sciences, Research Unit of General Practice; 5 Dialogos, Copenhagen, Denmark

**Keywords:** Intercultural medicine, indigenous, herbalists

## Abstract

**Background:**

In Uganda generally and in rural areas in particular, use of traditional medicine is a common practice, yet there remains lack of evidence on the overall utilization of traditional medicine and there are many aspects that remain unclear.

**Objective:**

To determine the use of traditional medicine and factors associated with this among the adults of Katikekile Subcounty in Moroto district.

**Methods:**

A descriptive cross-sectional study using quantitative and qualitative methods. Interviews among 323 respondents, and focus group discussions were carried out among village traditional birth attendants, village health team members, and traditional health providers.

**Results:**

Use of traditional medicine among the adults of Katikekile Subcounty was 68%. Usage was more prevalent among older people, and the majority of the adults used traditional medicine often as their first line-treatment for any illness. Herbs used for traditional medicines are usually locally available and free-of-charge. Long distance to health-facility based health care services, and medical fees contributed to the use of traditional medicine.

**Conclusion:**

Use of traditional medicine among adults of Katikekile Subcounty in Moroto in the Karamoja region in Uganda was high, and majority of the adults often used traditional medicine as first line-treatment. Both socioeconomic and health sector factors were associated with use of traditional medicine.

## Introduction

African Traditional Medicine (TM) is a holistic discipline that integrates indigenous use of herbs and spiritualism. It is based on indigenous experiences and knowledge, whether explicable or not, and used for general health purposes as well as for diagnosis, prevention or treatment of physical and mental illness^1^, and is widely used in sub-Saharan Africa including Uganda^2^. Diagnosis of illness is done using spiritual means, while treatment, consists of herbs. These herbs are not only believed to provide cure but have symbolic and spiritual significance. This differs from western medicine, which is, largely, scientific.

Previously, there has been a misbelief that the conception of disease in sub-Saharan Africa was embedded in “witchcraft”. None the less, the aetiology of illness in Africa is traditionally viewed from both supernatural and natural perspectives. TM practice has a wide scope covering treatment with herbal medicine^3^, bone setting, birth delivery and child health and mental health care among others. Some of the challenges with TM include is delay of treatment, patients not receiving the best available management, discontinuation of efficacious or interaction between traditional herbs and modern medicine^4^, ^5^. While there is potential danger in some traditional methods, TM healers serve an important role in health promotion, disease prevention and treatment^6^. In order to minimise distrust between modern and traditional health providers and to obtain cooperation and regulation, both traditional and modern health providers must acknowledge their areas of strengths and weaknesses 7,8.

Even though some traditional healers are being integrated into the national health system in Uganda to some extent, traditional medicine has not been fully incorporated, yet 60-80% of Ugandans visit traditional healers as their first line health care^9^, ^10^. It is also known that successful community-based intervention are based on mutual understanding between healthcare workers and community members^11^.

In a way, the use of traditional medicine has been researched to some extent, in order to establish the efficacy and efficiency of TM 12–14. None the less, most of the traditional medicines being used in Uganda have neither been studied nor regulated.

Some factors have been reported to be the reason for the widespread use of TM in sub-Saharan Africa. Accessibility of traditional health providers is, by and large, higher than modern western heath care providers. For example, the ratio of traditional health providers to the population is 1: 500 while that of for modern medical doctors is 1:40 000. We also know that most modern medical doctors are based in urban areas leaving people in rural communities with no choice but to consult traditional health providers in case of illness^8^. A recent review of factors affecting use of TM included low socioeconomic and educational status; while there were inconsistencies in gender, age and religious beliefs^15^.

It has been reported that older age and female gender is associated with increased use of TM 16, but there are conflicting data on how other factors such as educational level, religion and spiritual beliefs, marital status affect use of TM 17. However, the users of TM choose health practices that resonate with their beliefs about health^18^. It has, also, been reported that TM is more easily accessible mainly because of a multiplicity of users, the herbs normally grow in gardens or bushes close to the homes and hence their use is free of charge without any significant economic limitations 19.

Distance to the health facility has been found be related to the use of modern health facilities as well as modern medicine^1^, ^20^; In addition, health system factors, such as policies that control the use of TM, have a great influence on the use of TM. The attitude of health workers towards patients, and waiting time at health facilities before accessing health services, can attract patients or discourage them from using health services in modern health facilities 9. This might force them to use available alternatives such as TM 21.

While the use of TM in sub-Saharan Africa seems widespread, only a few studies have been made regarding factors related with TM use^15^. This is the main justification for further research to elucidate opportunities and challenges associated with use of TM

## Objectives

This study sought to determine the prevalence of traditional medicine use; and determine socio-economic as well as health system factors associated with use of traditional medicine in Katikekile Subcounty, Moroto district, Uganda.

## Methods

This was a descriptive cross-sectional study using quantitative and qualitative methods of data collection. It is a sequential explanatory design, where the data was collected in two consecutive phases. In the initial phase the researcher collected and analysed quantitative data. This was followed up by a second phase collecting qualitative data using a questionnaire based on the results from the first phase.

Multiple (simple random and probability proportionate to size) sampling methods were used since the population of interest comprised more subgroups. The number of participants from each subgroup was determined by their number relative to the entire population.

### Study site

The study took place in Katikekile Subcounty, Moroto district, Karamoja Region, Uganda. The population of the study area was approximately 1693 (Uganda national census 2002), and consist of Karimojongs, who traditionally run an agro-pastural or seminomadic lifestyle. The semiarid dry tree savanna district is largely rural with about 80% of the population living in rural areas and 20% of the people living in urban areas.

The subcounty was divided into four zones; east, west, north and south, corresponding to the administrative areas of the local councils (LC1). To obtain a non-biased number of subjects from each selected zone, a probability proportional to size method (the Kish Leslie formula of sample size determination^22^ was applied to estimate the number of respondents to interview from each zone based on the estimated population. The houses from each zone was numbered and randomly selected by balloting the house numbers. Then one adult from each household who was willing to participate and qualified per inclusion criteria in the study was interviewed. In all 323 respondents participated in the study.

The study was conducted during the month of June 2013. The population included all adults both adult men and women above 18 years of age. Participation in the study was voluntary and unpaid. Participants who had communication hindrances such as the deaf and mute were excluded from the study.

### Data collection

The researcher used researcher-administered questionnaires for collecting data. One questionnaire for collecting quantitative with closed ended questions and another questionnaire for collecting qualitative data including both open and close-ended questions. Use of TM was predominantly stated to be use of herbal medicine.

A pre-test of the questionnaires was done in another subcounty other than Katikekile to check and ensure its suitability, reliability and validity. Questions that were identified as not clear or irrelevant to the study were edited or omitted.

In addition to collecting quantitative data using the first questionnaire, focus group discussions were carried out to obtain qualitative data. Focus groups were formed by identifying both traditional and official healthcare providers in the study site. The focus groups thus included village traditional birth attendants, health officials at village level, known village health team (VHT) and traditional health providers/herbalists. Traditional birth attendants were purely women whereas the other groups were both male and female. Nine focus group discussions were carried out with 10 traditional birth attendants, 10 Health officials/VHT and 10 Traditional health providers/herbalists in the 3 villages. Each focus group discussion lasted for two and half hours. The focus group discussions were facilitated by the researcher herself, a local public health nurse with knowledge of the local area and population. As she was Karimojong, the discussions were carried out in the local language. The discussions were written down with arguments and quotes by the researcher and an assistant in English during the and after the focus group discussions.

During the focus group discussions, they were told to list all the common diseases and how they treat them in their community. The items gathered from free listing which were written in the separate cards, were sorted out by the group members to put in the piles according to the types of diseases and its treatment and on the other pile how easily they can access treatment and another the cost of treatment.

### Data analysis

To study the possible factors affecting use of TM the study participants were categorised by demographics into gender, age, level of education, religion and marital status. Socio-economic and health system factors such as accessibility of TM, cultural norms, distance to health centre, affordability of health care services, attitude of healthcare workers and monthly income were recorded as well. This to identify possible associations between these and use of traditional medicine. Only complete datasets were used in the analyses. Both crude and adjusted analysis were carried out using both univariable and multivariable logistic regression models to estimate the association (odds ratios) between the use of TM as outcome and the exploratory factors. The data is presented with 95% confidence intervals and thus a p-value of <0.05 was considered statistically significant. Computer software-Stata 16 was used. The qualitative data from the focus group discussion were analysed, where possible causal relationship between findings of the quantitative data were described. Arguments and opinions were then grouped into categories trying to give a picture of both the main trends among the focus group members and statements for either side from the main trend.

### Ethical consideration

Most participants were illiterate so verbal information about the study was given prior to collecting data for both the quantitative part and prior to the focus group interviews. A consent form signed either by writing or thump print. The study followed the guidelines provided by International Health Sciences University^23^ by seeking legal acceptance from the university in form of a letter of authorization from both the university and Katikekile Subcounty.

## Results

The study included 323 respondents. Data regarding socio-demographic of the respondents, health system factors and associations between these and usage of TM are presented in table 1. The use of TM was 221 of 323 (68.4%), and usage was more prevalent among older people, married people and people who believed there is no cultural influence for using TM. Long distance to the nearest health center or its services was also associated with higher use of TM. Of the respondents who used TM 31% reported they always used TM, 58% often and 11% rarely.

**Table 1:** Demographics, socio-economic indicators and health system factors associated with use of traditional medicine (TM) among people in Katikekile sub county, crude and adjusted Odds Ratios (OR)

		Use of traditional medicine?				
		
Variable		N	Yes	(%)	Crude OR (95% CI)	Adjusted OR (95%CI)
Total		323	221	(68.4)		
Sex	Male (ref)	117	80	(68.0)		
	Female	206	141	(68.4)	1.00 (0.66–1.63)	0.77 (0.43–1.37)
Age	<45 years (ref)	291	151	(66.6)		
	45 years	32	30	(93.7)	7.85** (1.84–33.5)	6.14* (1.35–27.94)
Level of education	Primary (ref)	171	108	(63.0)		
	Secondary	152	113	(74,3)	1.69* (1.05–2.73)	1.78* (1.01–3.12)
Religion	Christian (ref)	195	122	(62,6)		
	Muslims	128	99	(77.3)	2.04** (1.23–3.39)	1.65 (0.91–3.00)
Marital status	Not married (ref)	97	52	(53.6)		
	Married	226	169	(74.7)	2.57** (1.56–4.23)	2.38* (1.20–4.70)
Easy access to TM	No (ref)	174	108	(62.2)		
	Yes	149	113	(75.8)	1.92** (1.18–3.11)	1.79* (1.02–3.12)
Cultural influence to	Yes (ref)	144	88	(61.1)		
use TM?	No	179	133	(74.3)	1.84* (1.15–2.96)	3.04* (1.60–5.80)
Distance to health center?	≤ 1km (ref)	223	128	(57.4)		
	> 1km	100	93	(93.0)	9.86** (4.37–22.23)	7.75** (3.29–18.27)
Health care services	Affordable (ref)	183	120	(65.5)		
	Not Affordable	140	101	(72.1)	1.36 (0.84–2.20)	1.05 (0.59–1.87)
Attitude of health	Negative (ref)	283	188	(66.4)		
workers	Positive	40	33	(82.5)	2.38* (1.02–5.59)	1.93 (0.69–5.36)
Monthly income (Uganda Shilling)	≤ 50,000 (ref)	107	63	(58.9)		
	>50,000	216	80	(73.1)	1.90* (1.17–3.10)	N/A

* Statistically significant p<0.05

** statistically significant p<0.001

Ref: reference

Adjusted OR: adjustment includes all available variables in the analysis (data for monthly income from different dataset) 95% CI: 95% confidence intervals

High level of education was positively associated with increased use of TM, where 74% of people with a secondary school degree or higher used TM, versus 63% among respondents with a primary school degree or lower (adjusted p=1.78, CI 95% 1.01–3.12). And similarly, a high level of income was positively associated with increased use of TM, where 73% of people with a monthly income of 50,000 Uganda Shilling or more used TM, versus 59% among respondents with a monthly income of 50,000 Uganda Shilling or less (p=1.90, CI 95% 1.17–3.10).

A positive attitude of the health workers were found to be associated with an increased use of traditional medicine, however the study found that there was a predominantly negative attitude towards the patients at the health center and only 40/323 (12.4%) respondents find a positive attitude towards them when seeking assistance in the health facility.

The quantitative data and the positive associations were included in the focus groups to determine causality of the findings. The group discussion revealed causality of the analyses, and furthermore some interesting points were stated from the respondents listed below.

Attitude towards using TM was explained during the focus group discussions, where an elderly man (in favor of the TM) stated: The only unfortunate thing is that, some herbalists/healers are not ready to share the knowledge on the use of TM with the young people and as a result such indigenous knowledge on medicinal herbs that ought to be passed on from one generation to the next might soon be lost. However, one of the women (who apparently were against the use of TM) said: “one of the greatest challenges with the use of traditional medicine is that there is no mechanism that has been devised to measure the accurate dosage to be dispensed especially from the herbs used. As a result of using such herbs especially with the consultation of a traditional healer/herbalist, patients tend to delay to seek health-facility based medical care which encourages the advancement of the disease and since in most cases the patients have already used herbs, the risk of developing drug-resistant strains of infections is high”. Most herbs used for traditional medicines are within the homesteads, gardens or near-by bushes and are free-ofcharge, and thus quite accessible for the people to use. Easy access to TM seems to increase use of TM, which was consistent with the findings in the focus group discussions, where discussants agreed that, “availability of most of the basic traditional medicines within the homesteads increases accessibility to such herbs and since there is no cost involved; most residents easily opt for traditional medicine rather than modern drugs”.

One traditional healer stated: “although herbs are known to treat different illnesses, even I, myself, am not sure of the active components in most of these herbs and the mechanism by which these herbs work. Often herbs are given to the patient on a trial and error basis. The patients always hope that the traditional medicine works before they patient return to change to another type of herb”.

According to a Village Health Volunteer, “there are many factors which prevent residents from utilizing modern health care services..... but the most intent factor is affordability of the services and medicines at health facilities”. He adds, “...most of the residents find such medicines very expensive because of the high levels of poverty in addition to ignorance and limited knowledge on prevention of disease. Most of the herbs relieve pain yet not curing the actual illness. So, patients using TM are in most cases made to believe that they are fine and that the medicine is working. Especially when it relieves them of fevers and pain. Also, long waiting time and distances moved to the health facility, force patients to resort to TM which is readily available in the community”.

**Figure 1:**
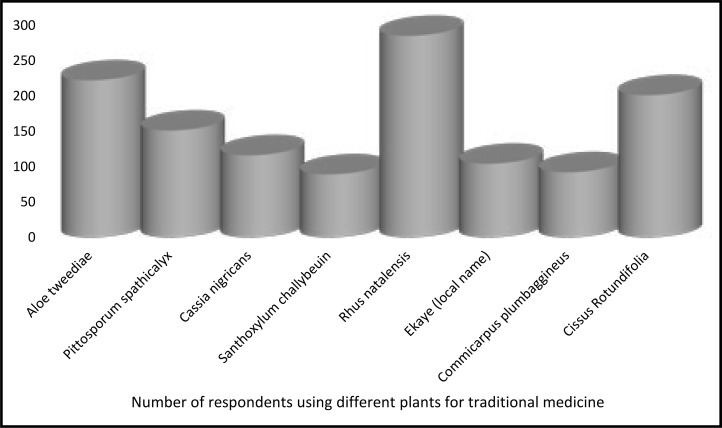
Examples of plants for traditional medicines used by respondents (in Latin names)

Examples of traditional medicines commonly used by the participants are shown in figure 1.

## Discussion

This study found that the prevalence of use of (TM) among the adults of Katikekile Subcounty was 68%, and that most of the adults used traditional medicine often as their first line-treatment for any illness. Socioeconomic factors like older age, higher educational level of education, high monthly income and being married were associated with higher use of TM. Also, health system factors like easy availability of TM and long distance to health facility were associated with high use of TM.

### Strengths and weaknesses

The results from this study should be interpreted with some caution taking potential weaknesses of the study into consideration. Participating in the study was voluntary which may have influenced who would participate rate and representativeness of the study population. As the researcher represents the health care system one might believe that some respondents would be more hesitant to tell whether they used TM. The result represents the usage of TM in one particular community, and the results are not necessarily likely to be generalizable to other parts of Uganda or rural areas.

Strengths of the study were that the researcher was from that culture herself, and understood the people, the language and culture. By using a sequential explanatory design, where the data was collected in two consecutive phases, firstly collecting and analysing quantitative data and secondly using results from the first phase to conduct focus group obtaining qualitative data add to the credibility of the results.

### Interpretation and comparing result

Our findings are in line with previously studies, where use of TM has been stated to be between 60–80% among Ugandans and often as their first line of treatment^9^, ^10^. Similarly, other studies document relatively high use of TM alone or in conjunction with modern medicine in Sub-Saharan countries, where approximately two thirds of users of fail to disclose this use to their healthcare providers^15^.

Socio-economic factors and use of traditional medicine Usage was more prevalent among older people. The age of TM users has previous been reported to younger (20–50 years) of age in urban or semiurban settings^24^,^25^ but in rural settings older people (>50 years) are more likely to use TM^26^, ^27^. That older people have higher use of TM could be related to both cultural issues, but also, that older people are more prone to suffer from medical conditions and hence require increased health care. In Katikekile, due to cultural influence and existence of cultural norms, which encourage the use of traditional medicine the youth are taught on how to use TM at an early age by their elders.

Married people and people who believed there is not a cultural influence for using TM were more likely to use this. Generally, TM users compared with non-TM users in Sub-Saharan Africa were more often reported to be married than not married^28^–^30^. Young people who are still single are most likely to be staying with their elderly parents, guardians or relatives and might be influenced by the practices of these significant others. Similarly, the use of TM among married people could be influenced by the backgrounds of the couple. Therefore, the influence of marital status on the use of traditional medicine lies in the background and influence of the spouse on the use of TM or conventional medicine so usage of TM among married people does not come as a surprise. Surprisingly we found that higher educational level of education was associated with higher use of TM. This contradicts observations from previous studies, where TM users are reported to have little or no formal education^24^-^27^, ^31^, though a similar observation has been reported as ours^32^. People with high level of education and high level of income might have more knowledge and possibility to care for themselves and thus invest in health more than people with less education and lower income. This includes use of TM. Using TM in our study might thus reflect an increased focus and concern of health-related issues among people with higher education rather than preference of type of health care (tradition versus modern). This is also like to be the case regarding that a high monthly income was associated with higher use of TM.

Also, health system factors like easy availability of TM and long distance to health facility were associated with high use of TM.

The influence of religion on the use of traditional medicine does not exactly prove to deter the use of different herbs since it does not clearly speak against it. In some societies where traditional medicine has been highly associated with witchcraft, Christianity and Islam tend to discourage the use of traditional medicine especially in cases where users go to shrines of traditional practitioners33. On the other hand, believers in traditional religions believe that conventional medicine is not in line with their faith and it is their gods who can guide the TM men to herbal medicine to treat specific diseases^33^.

### Health system and use of traditional medicine

Easy availability of TM was associated with high use of TM. This finding was backed up during the focus groups interviews, where it was stated, that most herbs used for traditional medicines were within the homesteads, gardens or near-by bushes and were free-of-charge, and hence quite accessible. This was unlike health-facility based health care services, which are normally distant from the homesteads and attract transport fare as well as medical fees.

Long distance to health facility were associated with high use of TM. The distance and the time it takes to travel and the costs such as transport fare and medical fees often deter patients from going to the facilities to access modern medicines^1^, ^20^. This influences most patients to use traditional medicine as their first-line treatment for any illness and only go to the health facility when the illness has advanced or when more complications have developed. Limited access to health care services at the health facility where most patients are delayed due to congestion especially in public health centre often results into long waiting time. Yet patients and the people who escort them to the health facility usually have a lot of domestic and commercial work which they have to accomplish back home.

While the negative attitude of health workers might discourage patients from coming to health facilities for conventional medicine in preference for TM, there is increasing focus on intercultural medicine to overcome barriers among indigenous people^34^. Hence, patients who have had bad experiences where they have been abused or assaulted by health workers would not easily want to return to the health facility for any service and would go for TM, which is normally administered by a close family member or relative rather than visiting an abusive health worker. It is curios that the current study showed the opposite of this. However, there are challenges involved in the use of TM for both the individual users and the entire health system at large. Such challenges include lack of mechanism for measuring accurate doses when using herbs; limited scientific research to establish the effectiveness and efficacy; the risk of developing drug-resistant restrains germs, and limited disease notification rate at both regional and national levels. In recognition of the importance of TM, Uganda recently passed a bill intended to provide a regulatory framework for traditional herbalists and integrate TM in the healthcare system^35^.

There is minimal scientific research on most of the traditional medicines that are commonly being used so as to establish whether such medicines or herbs contain the curative ingredients necessary for the treatment of the diseases they are meant to cure^12^, ^13^, ^36^. Despite the benefits of using traditional medicines in providing an alternative form of health care medication, the high prevalence of TM use poses challenges to the health care system at both national and regional levels. Most of the illnesses are not properly diagnosed, which could result into wrong treatment and TM could have devastating effects or delay of modern treatment, which then could increase morbidity and become fatal.

## Conclusions

The use of Traditional Medicine among adults of Katikekile Subcounty in the Karamoja region of Uganda was high but use of TM compared to modern medicine is not a matter of ‘either-or’, but rather ‘both-and’. None the less, use of TM is more prevalent among older people, married couples and those who live far from official health facilities. Most herbs used for traditional medicines are grown near-homesteads and are, by and large, free-of-charge. Hence they are quite accessible compared long distance to health facilities which a facilitator for using TM.
